# Donation from Prof. Henry S. Chase

**Published:** 1877-07

**Authors:** 


*Donation from Prof. Henry S. Chase.*—The Baltimore College of Dental Surgery has lately received one hundred valuable microscopical specimens of the human teeth, beautifully prepared and ready mounted for investigation with the microscope, all of them being the work and gift of Prof. Henry S. Chase, D. D. S., M. D., of St. Louis, Mo.

